# Vapor phase mediated cellular uptake of sub 5 nm nanoparticles

**DOI:** 10.1186/1556-276X-7-212

**Published:** 2012-04-11

**Authors:** Tetiana Serdiuk, Vladimir Lysenko, Valery A Skryshevsky, Alain Géloën

**Affiliations:** 1 University of Lyon, Nanotechnology Institute of Lyon (INL), UMR-5270, Centre National de la Recherche Scientifique, Institut National des Sciences Appliquées de Lyon, Villeurbanne, F-69621, France; 2 Institute of High Technologies, Taras Shevchenko National University of Kyiv, 64, Volodymyrs'ka Str, 01601 Kyiv, Ukraine; 3 University of Lyon, CarMeN Laboratory, Unité Mixte de Recherche, UMR INSERM Institut National de la Santé et de la Recherche Médicale, 1060 INSA de Lyon, France

**Keywords:** nanoparticles, cell labeling, fluorescence, contamination

## Abstract

Nanoparticles became an important and wide-used tool for cell imaging because of their unique optical properties. Although the potential of nanoparticles (NPs) in biology is promising, a number of questions concerning the safety of nanomaterials and the risk/benefit ratio of their usage are open. Here, we have shown that nanoparticles produced from silicon carbide (NPs) dispersed in colloidal suspensions are able to penetrate into surrounding air environment during the natural evaporation of the colloids and label biological cells via vapor phase. Natural gradual size-tuning of NPs in dependence to the distance from the NP liquid source allows progressive shift of the fluorescence color of labeled cells in the blue region according to the increase of the distance from the NP suspension. This effect may be used for the soft vapor labeling of biological cells with the possibility of controlling the color of fluorescence. However, scientists dealing with the colloidal NPs have to seriously consider such a NP's natural transfer in order to protect their own health as well as to avoid any contamination of the control samples.

## Background

During the last decade, the biomedical research field has been identified as one that can greatly benefit from the advancement in nanotechnologies. In particular, the use of various kinds of nanoparticles (example: metallic, metal oxides, semiconductors, silica, etc.) in biotechnology merges successfully the fields of materials science and biology [1]. It has been found that different nanoparticles (NPs) can enter living cells, providing specific tagging of cell components. Nowadays, NPs are preferred to be used for multicolor cell imaging due to their unique properties, such as size and composition-induced tunable spectral characteristics, narrow emission spectra, high quantum yield, and low photobleaching [2]. In particular, II-VI NPs [3,4] and silicon NPs [5] are among the most extensively studied. We have already presented studies which implemented NPs produced from SiC as fluorescent agents for living cell imaging, similar to what our colleagues did [6,7]. We have also found preferential toxic effect of these NPs on cancer cells, which was described in detail in a previous work [8].

Although the potential of nanoscale objects in biology is tremendous, a number of questions concerning the safety of nanomaterials and the risk/benefit ratio of their usage remain open. Thus, nanotoxicology refers to the study of potential negative effects coming from the interaction between nanomaterials and biological objects [9,10]. Toxicity of NPs is studied from NPs in suspensions [11] except for the studies of lung toxicity where NPs are studied as aerosols [10,12].

In the following work, we show that even when NPs have been initially dispersed in liquids, they may be easily transported into the surrounding air environment at relatively long distances due to the natural evaporation of the liquids at room temperature. Such an innate transfer of NPs from the originally colloidal suspension into an aerosol state may easily lead to a significant labeling of any biological system, although it is separated from the colloidal source of NPs, by a gas phase. These results should be taken into account in order to (a) use them for soft cell labeling through the vapor phase, (b) avoid any contamination of control samples and (c) improve health protection of users.

## Methods

Fluorescent NPs were formed by means of electrochemical anodization of a low-resistivity grade (< 1 Ω) bulk SiC polycrystalline wafer (NPs) [6]. The etching process took place for 2.5 h at a current density of 25 mA/cm^2 ^using a 1:1 HF (50%)/ethanol electrolyte. After the etching, a highly porous network constituted by interconnected SiC nanocrystals was formed. The ultraporous layer was washed several times with deionized water then naturally dried in ambient air, removed from the wafer and ground to powder. To obtain colloidal suspensions of NPs, the powder was dispersed in 1:1 deionized water/ethanol mixture or Dulbecco's Modified Eagle's Medium (DMEM) buffer for mammalian cell investigations, and then large NP crystallites were removed by centrifugation at 10,000 g for 5 min. These suspensions contained only small-sized (< 3 nm) and uniformly dispersed NPs. Typical TEM images, dynamic light scattering size distributions and zeta-potential measurement of which had already been shown in the study by Botsoa et al. [6].

The inner layer of a fresh thin onion epidermis was peeled off. The onion peels were neatly spread in the center of a glass slide in a drop of water. The glass slide was then mounted on a mobile holder situated above a colloidal suspension (2 mL, surface area 4 cm^2^) containing the NP suspension (1 g/L). Height origin was fixed at the suspension surface. At a fixed height value, the onion epidermis samples were exposed to the suspension vapors for different periods of time varying from 3 to 17 h. Depending on exposure time, evaporated volume was changing from 0.5 to 1.5 mL. All experiments were carried out at a temperature of 21°C, humidity of 60% and vapor pressure of 1,018 hPa, without air mixing. Speed of convection flow was equal to 0 m/s. The manipulations were performed under two different conditions: (a) in open air and (b) in confined air with the whole system being covered by a cap. After a certain exposure time, the onion peels were intensively rinsed in deionized water to remove the NPs deposited onto the epidermis surface and unincorporated inside the epidermal cells. Control manipulations were carried out with the use of a pure suspension without the NPs.

Suspension containing NPs was also added to the 3 T3-L1 fibroblasts (American Type Culture Collection, Manassas, VA, USA). The cells were grown on glass substrates in DMEM supplemented with 10% newborn calf serum, 4 mM glutamine, 4 nM insulin (Actrapid Human Torrent Pharmaceuticals Ltd., Ahmedabad, India; Novo Nordisk A/S, Bagsvaerd, Denmark), 10 mM HEPES, 25 μg sodium ascorbate, 100 IU penicillin, 100 μg streptomycin and 0.25 mg/L amphotericin B at 37°C in a water-saturated atmosphere with 5% CO_2 _in a Heraeus incubator (BB16; Thermo Scientific, Brebières, France). The cells have been incubated for 15 h in the presence of NPs (with concentration of 0.8 g/L in suspension in DMEM). Some 3 T3-L1 samples were cultured in DMEM without NPs but, in air contact, were allowed with wells containing NP suspensions. After a certain incubation time, cells were rinsed twice in pure ethanol to eliminate NPs from the environment and to fix cells.

Finally, the samples were observed under a fluorescence microscope (Leica DMI 4000B, Leica Microsystèmes SAS, NANTERRE, France) using the following filter combination: UV/violet excitation band at 2.92 to 3.5 eV with an observation spectral range of < 2.64 eV. Leica EC 3 camera (Leica Camera Sarl, Saint Jorioz, France) was used.

For each image, luminosity per cell was calculated by summing intensities (in a range from 0 to 255) of each pixel in the picture and dividing this result by the number of fluorescent cells in the picture. The MathWorks Matlab R2009b and Simulink software (Torrance, CA, USA) were used for image analyses in order to quantify luminosity per one cell.

## Results and discussion

**Figure 1:**
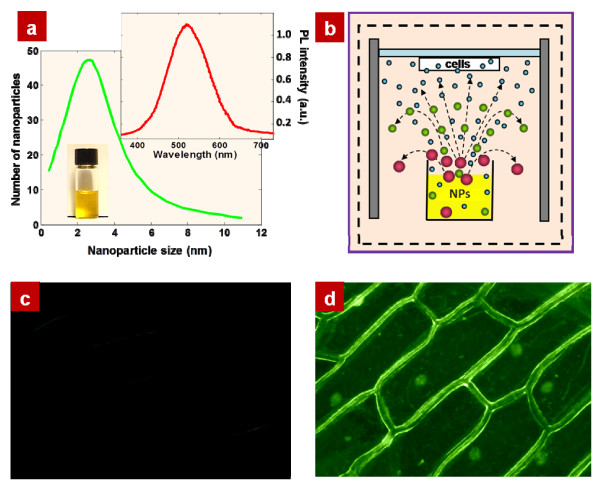
**Size distribution of the NPs (a)**. Inset shows a typical photoluminescence spectrum of NPs obtained from 3 C-SiC. (**b**) Schematic view of the experimental set-up. Onion cells are mounted on a mobile holder situated above a colloidal suspension containing the fluorescent NPs. Fluorescence images of the onion epidermal cells exposed to the suspensions without (**c**) and with (**d**) NPs.

Typical size distribution of NPs obtained from the 3 C-SiC is presented in Figure 1a. As one can see, the majority of the NP dimensions are below 5 nm, with the most probable size value being around 2.5 nm. The inset in Figure 1a shows a typical photoluminescence spectrum of the NPs dispersed in aqueous suspensions under ultraviolet excitation. Since the mean size value of the NPs shown in Figure 1a is smaller than the Bohr's diameter of the exciton in bulk SiC substrate (approximately 5.4 nm), they exhibit highly efficient luminescence with energies higher than the bandgap energy of the bulk SiC substrate due to spatial and quantum confinement effects [13,14]. An example of a daylight photo of the centrifuged optically homogeneous colloidal suspension of the NPs used in our work is also shown in Figure 1a. Experimental samples were fixed above the NP suspension, as it is schematically shown in Figure 1b at different height.

Typical fluorescence images of the onion epidermal cells exposed to the evaporating suspensions without and with NPs are shown in Figure 1c, d, respectively. As one can see, no fluorescence is visible in the absence of NPs (Figure 1c), while the cell structure of the fluorescent onion epidermis consisting of large, rectangular cells may be easily recognized in the presence of NPs (Figure 1d). The latter is due to the fluorescent labeling of the onion cells by the NPs originating from the colloidal suspension vapors. Indeed, water molecules leaving the suspension during its natural evaporation carry away the very light NPs into the surrounding air. Thus, initially colloidal NPs are transferred from liquid to vapor environment, reaching the cells, entering inside and tagging them. Similar to the fluorescent labeling of the 3 T3-L1 fibroblast cell culture reported earlier [6], a strong heterogeneous distribution of the fluorescent intensity through the onion cells may be observed. Indeed, one can notice that the external cell membranes and nuclei are preferentially tagged by the fluorescing NPs. The cytoplasm of the onion cells contains some small fluorescent spots as well, but their number is low, and the corresponding fluorescence intensity is weak in comparison to the fluorescence level of the cell membranes and nuclei.

**Figure 2:**
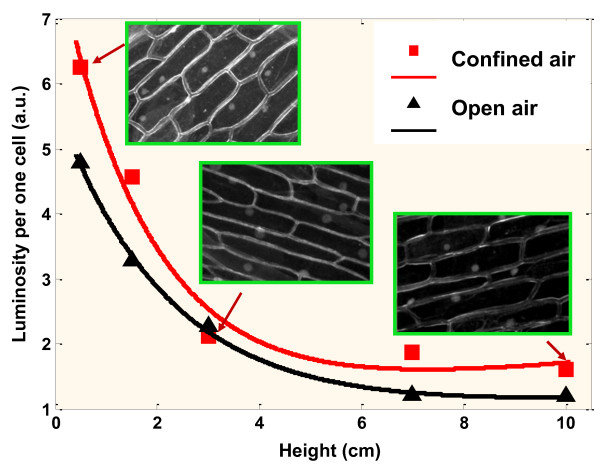
**Relationship between the integrated luminosity per one cell and vertical distance from the NP source**. Dependence of the integrated luminosity per one cell on the vertical distance between the cell holder and the suspension surface for two cases: open and confined air. Three inserted black-and-white fluorescent pictures illustrate some experimental points.

Figure 2 illustrates the relationship between the integrated luminosity per one cell and vertical distance from the cell holder to the suspension surface (see relationship between the integrated luminosity per one cell and horizontal distance from the NP source in Figure S1 in Additional file 1). The higher the holder position is, the weaker the cell fluorescence intensity obtained, suggesting that the number of the NPs reaching the onion peels decreases significantly with the distance between onion cells and the colloidal suspension of NPs. Three inserted pictures of fluorescence give a qualitative representation of the fluorescence intensity reduction according to the distance of the onion cells to the colloidal suspension of NPs. In addition, the curve representing the fluorescence of onion cells in function to the distance is nonlinear for both open and confined air. Obviously, cell luminosity is generally higher in the case of confined air through all the range of height values.

**Figure 3:**
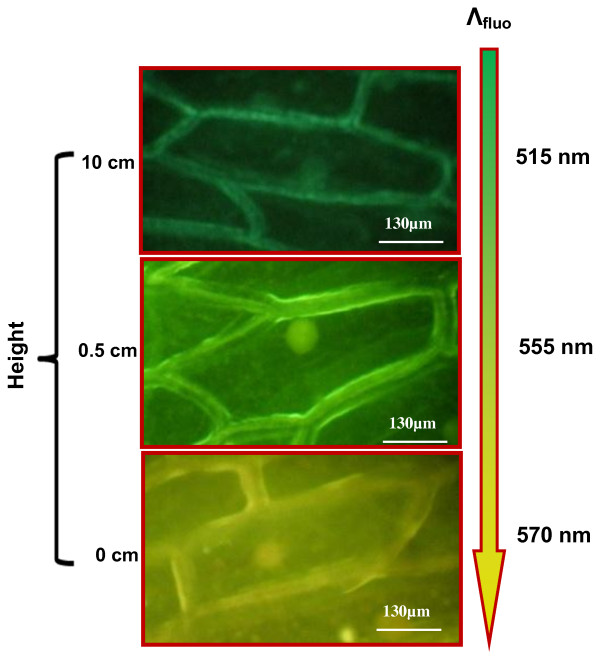
**Variation of fluorescence color of the onion cells labeled by the NPs via vapor phase**. Fluorescence images of the onion cells situated at different vertical distances from the colloidal suspension of NPs.

Figure 3 shows the variation of fluorescence color of the onion cells labeled by the NPs via vapor phase as function of the distance to the colloidal NP suspension. The cell fluorescence is continuously red-shifted when the distance between the onion cells and the NP suspension surface decreases. Taking into account that due to the quantum confinement effect, the smaller the NPs are, the shorter their fluorescence wavelength is, it can be concluded that, obviously, smaller nanoparticles are much easier to be transported in vapor phase. In particular, according to the low mean of Bohr's diameter (5.4 nm), even very small changes of NP diameter provoke strong shift of photoluminescence maximum position.

**Figure 4:**
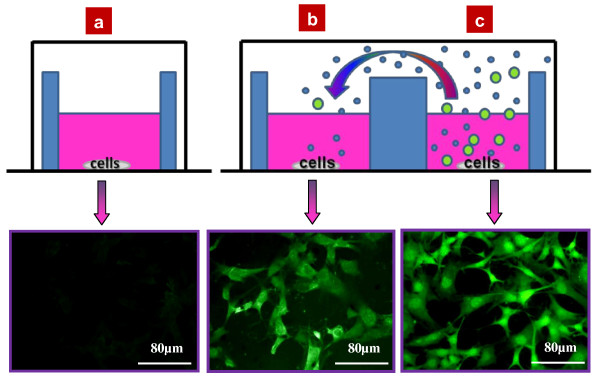
**Fluorescence images of the 3T3-L1 fibroblast cells**. Isolated (**a**) and non-isolated (**b**) from the air environment of a cell culture, (**c**) labeled with the fluorescent NPs.

**Figure 5:**
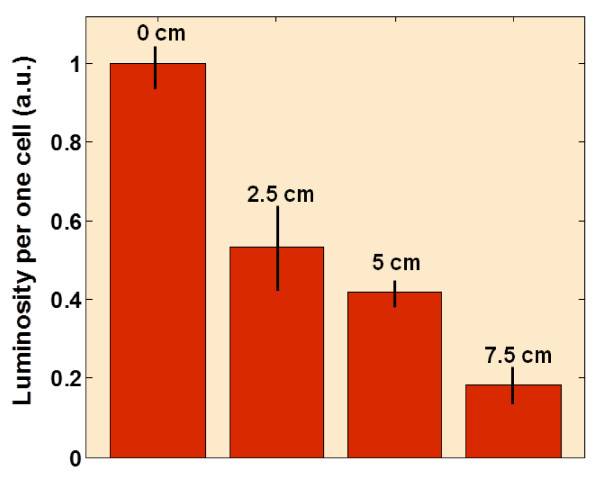
**Dependence of the integrated luminosity per one cell**. The cell cultures were exposed to NPs via air phase on the different horizontal distance between them and the cell culture containing the colloidal fluorescent NPs.

Another essential application of the present observation concerns the contamination of control samples. Figure 4 shows that a control cell sample (3 T3-L1 mouse fibroblasts) situated close to a well containing colloidal NP suspension can be easily contaminated through the vapor phase. Indeed, as one can see, the corresponding fluorescent pictures of both cell cultures, control without NPs and experimental with NPs, are characterized by almost the same fluorescent intensities, while a reference completely isolated from all the colloidal NP suspensions remains fully unlabeled. Dependence of the fluorescent luminosity per one cell in function of the horizontal distance between the cell culture containing the colloidal fluorescent SiC NPs and the cell cultures labeled via air is shown in Figure 5. Intensity of the fluorescent labeling coming from the surrounding air containing NPs is inversely dependent to the distance from the intentionally labeled cell culture wells. A natural gradual size-tuning of SiC NPs was observed in dependence of the distance from the NP source. This results in a progressive shift of fluorescence color of labeled cells to the red region with the distance decrease. That effect may be used for the soft vapor labeling of biological cells, allowing for the control of their color. However, to avoid any parasitical contamination of experimental samples via vapor phase, one has to spatially isolate the cell cultures containing colloidal NP suspension.

## Conclusion

In summary, the present study demonstrates that NPs in colloidal suspensions can be transported in the environment through evaporation to rather long distances and can reach biological targets. In particular, observed results need future development of deep theoretical understanding. The main conclusion is to warn scientists dealing with the colloidal NPs to seriously consider air contamination in order to take care of their own health protection as well as to avoid any contamination of their control samples sharing air environment with the colloidal suspensions containing dispersed NPs.

## Competing interests

The authors declare that they have no competing interests.

## Authors' contributions

TS performed the mammalian sample growth, nanoparticle fabrication, measurements and data analysis. VL performed the general data analysis and contributed to the discussion of the obtained data. VAS also contributed to the discussion of the data analysis. AG also performed the mammalian sample growth and coordinated the project and data analysis. All authors participated in writing the manuscript and approved its final version.

## Supplementary Material

Additional file 1 **Figure S1**. Fluorescence images of the onion cells situated at different horizontal positions (a-6 cm, b-9 cm, c-12 cm, d-16 cm). Click here for file

## References

[B1] De M Ghosh PS Rotello VM Application of nanoparticles in biology Adv Mat 2008 20 1 17

[B2] Bruchez M Moronne M Gin P Weiss S Alivisatos AP Semiconductor nanocrystals as fluorescent biological labels Science 1998 281 2013 2016 9748157 10.1126/science.281.5385.2013

[B3] Chen L Mccrate JM Lee J Li H The role of surface charge on the uptake and biocompatibility of hydroxyapatite nanoparticles with osteoblast cells Nanotech 2011 22 105708 10.1088/0957-4484/22/10/105708 PMC3144725 21289408

[B4] Schaeublin N Braydich-Stolle L Schrand A Miller J Hutchison J Schlager J Hussain S Surface charge of nanoparticles mediates mechanism of toxicity Nanoscale 2011 3 410 420 10.1039/c0nr00478b 21229159

[B5] Alsharif NH Berger CEM Varanasi SS Chao Y Horrocks BR Datta HK Alkyl-capped silicon nanocrystals lack cytotoxicity and have enhanced intracellular accumulation in malignant cells via cholesterol-depended endocytosis Small 2009 5 2 221 228 19058285 10.1002/smll.200800903 PMC2962801

[B6] Botsoa J Lysenko V Géloën A Marty O Bluet J-M Guillot G Application of 3C-SiC quantum dots for living cell imaging Appl Phys Lett 2008 92 173902 10.1063/1.2919731

[B7] Fan J Li H Jiang J So LKY Lam YW Chu PK 3C-SiC nanocrystals as fluorescent biological labels Small 2008 4 1058 10.1002/smll.200800080 18618492

[B8] Mognetti B Barberis A Marino S Di Carlo F Lysenko V Marty O Géloën A Preferential killing of cancer cells using silicon carbide quantum dots J Nanosci Nanotechnol 2010 10 7971 7975 10.1166/jnn.2010.3049 21121285

[B9] Brayner R The toxicological impact of nanoparticles Nano Today 2008 3 48 55

[B10] Singh N Manshian B Jenkins GJ Griffiths SM Williams PM Maffeis TG Wright CJ Doak SH The DNA damaging potential of engineered nanomaterials Biomaterials 2009 30 3891 3914 10.1016/j.biomaterials.2009.04.009 19427031

[B11] Lewinski N Colvin V Drezek R Cytotoxicity of nanoparticles Small 2008 4 26 49 10.1002/smll.200700595 18165959

[B12] Warheit DB Nanoparticles: health impacts? Mat Today 2004 7 32 35

[B13] Fan JY Chu PK Group IV nanoparticles: synthesis, properties, and biological applications Small 2010 6 2080 2098 10.1002/smll.201000543 20730824

[B14] Zakharko Yu Botsoa J Alekseev S Lysenko V Bluet J-M Marty O Skryshevsky VA Guillot G Influence of the interfacial chemical environment on the luminescence of 3C - SiC nanoparticles J Appl Phys 2010 107 013503 10.1063/1.3273498

