# Supplementary material for: Vapor phase mediated cellular uptake of sub 5 nm nanoparticles
Source: Nanoscale Res Lett. 2012 Apr 11;7(1):212. doi: 10.1186/1556-276X-7-212 (PMC3368743; doi:10.1186/1556-276X-7-212)
Supplement: Additional file 1 — Figure S1. Fluorescence images of the onion cells situated at different horizontal positions (a-6 cm, b-9 cm, c-12 cm, d-16 cm). [file 1556-276X-7-212-S1.DOC]

###### *Supporting information*

Figure S1 illustrates dependence of the integrated luminosity per one cell on the horizontal distance between the cell holder and the NPs suspension surface. Increasing of the distance from colloidal solution to sample provoke decreasing of vapour mediated NP labelling. Luminosity per one cell for control cells that had not any contact with NPs was taken as relative unite. Figure S1 a, b,c, d illustrate horizontal labelling of sample through the vapour at the distances 6 cm, 9 cm, 12 cm and 16 cm, correspondently.


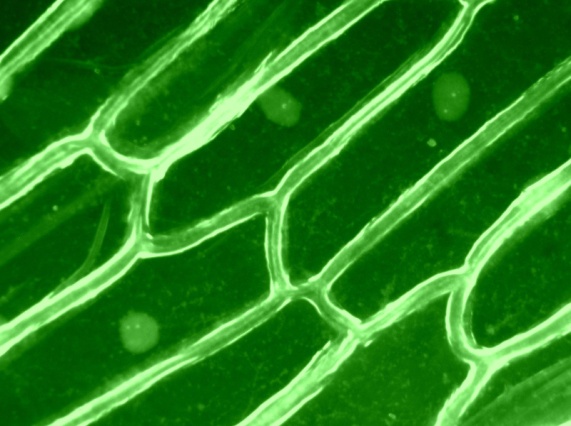


**a**

**Luminosity per one cell (a.u.)**


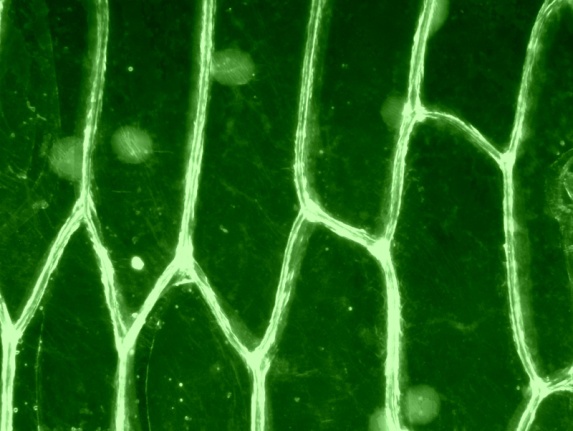


**b**

**Luminosity per one cell (a.u.)**


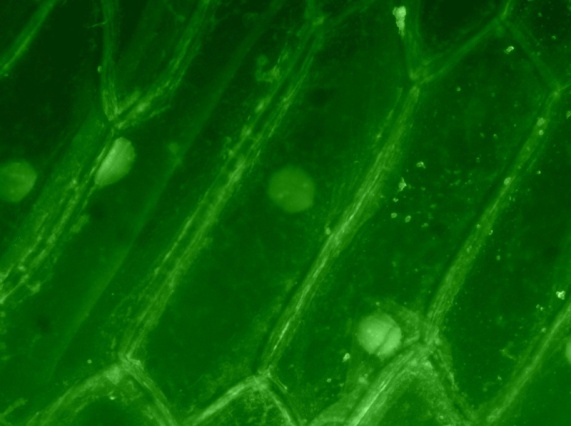


**c**

**Luminosity per one cell (a.u.)**


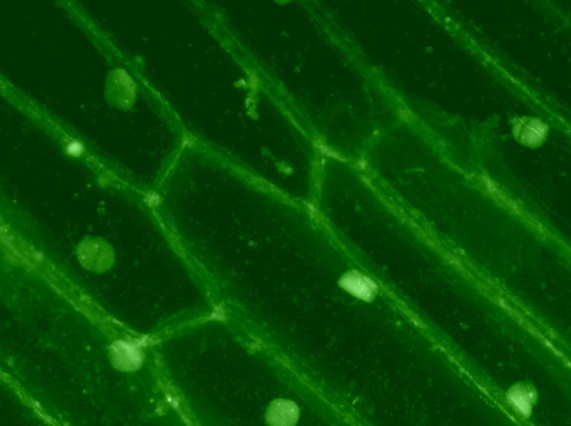


**d**

**Luminosity per one cell (a.u.)**

**100µm**

**100µm**

**100µm**

**100µm**

Figure S1. Fluorescence images of the onion cells situated at different horizontal positions (a-6 cm, b-9 cm, c-12 cm, d-16 cm).
